# Separate lifetime signatures of macaque S cones, M/L cones, and rods observed with adaptive optics fluorescence lifetime ophthalmoscopy

**DOI:** 10.1038/s41598-023-28877-6

**Published:** 2023-02-11

**Authors:** Khang T. Huynh, Sarah Walters, Emma K. Foley, Jennifer J. Hunter

**Affiliations:** 1 Department of Biomedical Engineering, University of Rochester, Rochester, NY 14627 USA; 2 Center for Visual Science, University of Rochester, Rochester, NY 14642 USA; 3 Currently with IDEX Health & Science, West Henrietta, NY 14586 USA; 4 The Institute of Optics, University of Rochester, Rochester, NY 14627 USA; 5 Flaum Eye Institute, University of Rochester, Rochester, NY 14642 USA

**Keywords:** Retina, Adaptive optics, Biomedical engineering

## Abstract

In the retina, several molecules involved in metabolism, the visual cycle, and other roles exhibit intrinsic fluorescence. The overall properties of retinal fluorescence depend on changes to the composition of these molecules and their environmental interactions due to transient functional shifts, especially in disease. This behooves the understanding of the origins and deviations of these properties within the multilayered retina at high lateral and axial resolution. Of particular interest is the fluorescence lifetime, a potential biomarker of function and disease independent of fluorescence intensity that can be measured in the retina with adaptive optics fluorescence lifetime ophthalmoscopy (AOFLIO). This work demonstrates the utility of the phasor method of analysis, an alternate approach to traditional multiexponential fitting, to evaluate photoreceptor two-photon excited AOFLIO data and separate them based on functional differences. Phasor analysis on fluorescence lifetime decay data allowed the repeatable segregation of S from M/L cones, likely from differences in functional or metabolic demands. Furthermore, it is possible to track the lifetime changes in S cones after photodamage. Phasor analysis increases the sensitivity of AOFLIO to functional differences between cells and has the potential to improve our understanding of pathways involved in normal and diseased conditions at the cellular scale throughout the retina.

## Introduction

The in vivo fluorescence properties of the retina provide a gateway into understanding molecular pathways that are compromised in diseases. Sources of autofluorescence include key molecules of the visual cycle and cellular metabolism such as all-*trans*-retinol, nicotinamide adenine dinucleotide phosphate (NADH), and oxidized flavin adenine dinucleotide (FAD)^1,2^. One property of fluorescence, the fluorescence lifetime (the rate at which fluorescence emitted from an excited fluorophore decays), is affected by factors that can change in disease including the relative concentrations of the excited fluorophores, pH, and interactions with enzymes^3–6^. More generally, cells with divergent biochemical pathways because of functional differences will likely have unique lifetimes. The fluorescence lifetime of the retina can be measured in vivo at a subcellular scale with two-photon excited adaptive optics fluorescence lifetime imaging ophthalmoscopy (AOFLIO), which has the potential to be a minimally invasive tool that can further our understanding of retinal biology and physiology^7,8^.

Previously, we performed two-photon excited AOFLIO of photoreceptors in the living macaque^8^. Using a biexponential fit of the fluorescence decay curves, we found that cone photoreceptors have longer mean lifetimes than rod photoreceptors. Functional differences such as photopic sensitivities of cones and rods^9^ might give rise to the longer lifetimes of cones. On the other hand, it was unclear whether the three cone classes, short-(S), medium-(M), and long-(L) wavelength sensitive cones, different in their spectral sensitivities, metabolism, and other properties^10–12^, had unique lifetimes. The mean fluorescence lifetimes of individual cones were unimodally distributed, which suggested no lifetime difference between the cone classes^8^.

An alternative approach to analyzing fluorescence lifetime decay data is phasor analysis^13,14^. The Fourier transform of the decay curve for each region of interest is evaluated at the same frequency and represented as Cartesian coordinates. This graphical approach facilitates visual comparisons between the lifetimes of various tissue types where cells with different fluorophore compositions will aggregate in different locations in the two-dimensional space even if they have similar mean lifetimes^13^. The phasor approach requires no prerequisite knowledge of fluorophores and avoids many of the subjective criteria required to set up exponential decay models including the number of fit components (analogous to the number of fluorophores) or the choice of fitting algorithm. Phasor analysis may even more robustly than multiexponential fitting quantify the relative contributions of fluorophores and make predictions about how vital functional pathways in the retina, such as metabolism or the visual cycle, shift in dysfunction^13,15,16^. Some applications of phasor analysis show potential in investigating the lifetime changes in local dysfunction in healthy and diseased retina^16,17^.

It is possible that subsets of cones can be distinguished by phasor analysis due to functional differences that are not captured by lifetime parameters extracted from multiexponential fitting. In this paper, we complement our previous work on AOFLIO in nonhuman primates as a tool for understanding the endogenous fluorescence of the retina with application of the phasor method of analysis. We investigate the separability and repeatability of distinct AOFLIO signatures of S cones, M/L cones, and rods. The identities of S cones were confirmed by a selective S cone damage paradigm developed by Schwarz et al.^18^.

## Results

### Two distinct clusters of cones and one cluster of rods are distinguishable by phasor analysis

To test whether cones and rods had different phasor signatures, we measured the fluorescence lifetimes of non-human primate photoreceptors. A custom-built adaptive optics fluorescence lifetime imaging ophthalmoscope measured the fluorescence intensity and lifetime decays at a cellular scale in the living retina^8,19^. A single 3 mW exposure (~ 877 J/cm^2^) was delivered to 45 retinal locations across 3 macaques (“initial exposure”). At each location, the fluorescence lifetime decay curve was measured for every image pixel. Using the fluorescence intensity image, individual cones were manually encircled to create multiple masks to analyze individual cones and the rod regions. A phasor coordinate was calculated for the sum of all decay curves within each mask. The cone phasor coordinates appeared to aggregate into two distinct groups, so a Gaussian mixture model (GMM) was applied to cluster the coordinates.

**Figure 1:**
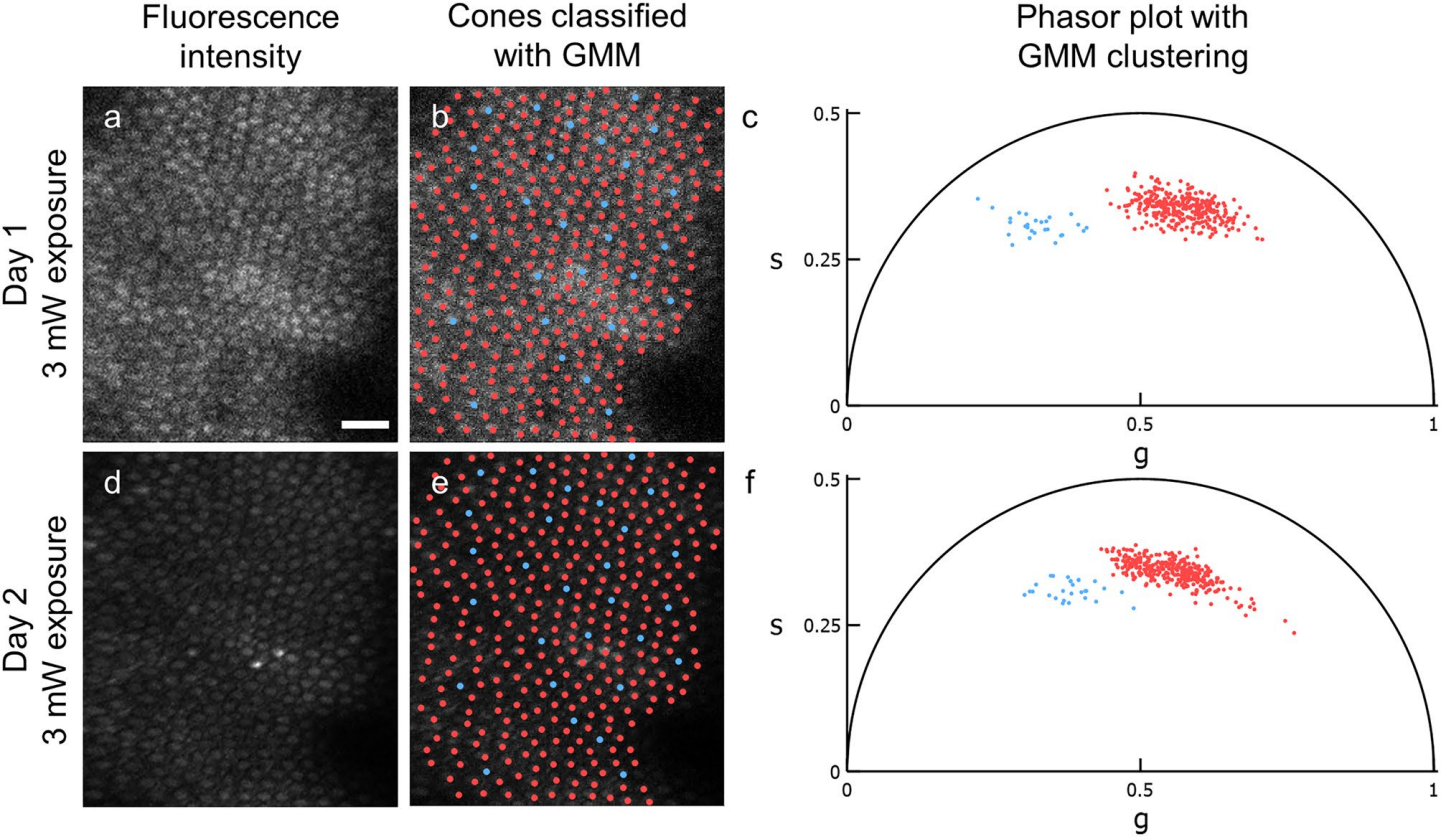
Repeatability of AOFLIO and phasor in partitioning the same cones. Representative fluorescence lifetime data acquired with a low-level exposure (3 mW, 877 J/cm^2^) taken on two different days at the same location (i.e., initial exposure (**a–c**) and Paradigm 1 (**d–f**)). In each fluorescence intensity image (**a,d**), the same 348 cones were marked (**b,e**) in red or blue according to their GMM cluster assignments in the phasor plots (**c,f**). Each phasor coordinate represents one cone. In both exposures, the same 28 cones, representing 8.1% of all cones, were assigned to Cluster 1. Cones obscured by the blood vessel shadow in the right side of the image were not marked. Scale bar represents 20 μm.

**Figure 2:**
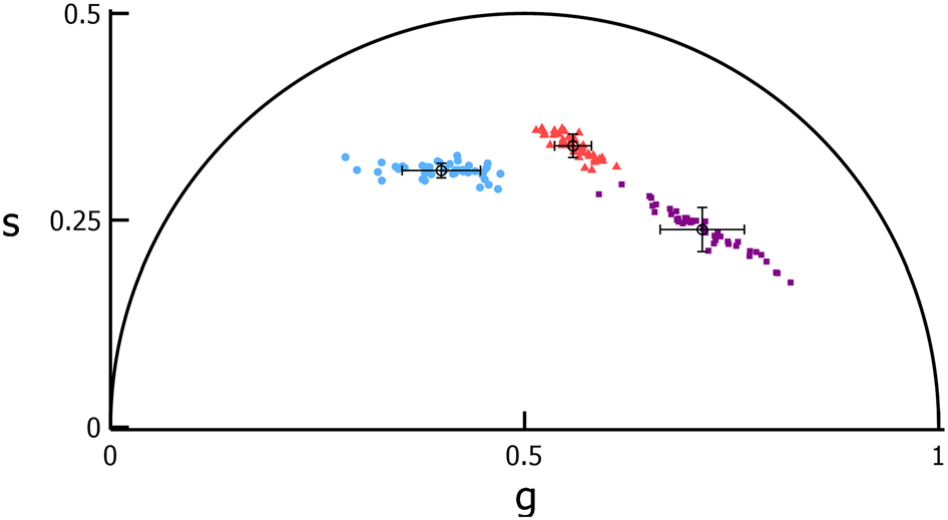
Grouping of photoreceptor phasor coordinates into distinct clusters. Average phasor coordinates of two cone clusters (“Cluster 1”, blue circles; “Cluster 2”, red triangles) and one rod cluster (“Cluster 3”, purple squares) for 43 initial exposure locations in 3 monkeys. Within each cluster, one point represents one cone cluster within an image.

Phasor analysis of cone decay curves and GMM clustering revealed two distinct clusters of cones, a smaller one closer to the origin (“Cluster 1”) and a larger one to the right (“Cluster 2”). As shown in Fig. 1a–c for a single location, when the cones in each cluster were mapped back onto the intensity image, the Cluster 1 cones consistently labeled a semi-crystalline mosaic; these cones were rarely adjacent to other Cluster 1 cones. Figure 2 shows the average phasor coordinates for the two clusters of cones classified by GMM and the rod regions at 43 of 45 initial exposure locations. The phasor coordinates for two locations (referred to in this manuscript as locations A and B) did not appear to aggregate into more than one distinct cluster; these locations were excluded from the GMM analysis. The decay curves of the arbitrarily defined rod regions manifest as a third cluster closest to (1,0) (“Cluster 3”). A significant difference of mean coordinates was observed between Clusters 1 and 2 ($p<$ 0.00005), Clusters 1 and 3 ($p<$ 0.00005), and Clusters 2 and 3 ($p<$ 0.00005) using Hotelling’s T^2^ test. Supplementary Table S1 (“Initial exposure”) provides the mean phasor coordinate for each cluster across all initial exposures.

Supplementary Table S2 (rows labeled “Initial exposure”) provides the number of cones marked and clustered by GMM for all initial exposures across all locations. Out of 12,502 cones marked, GMM identified 1467 cones which fell into Cluster 1. In two of 43 locations with two distinct clusters (locations C and D), the Cluster 1 cones identified by GMM were not distributed across the entire image. One example is shown in Supplementary Fig. S1 where the Cluster 1 cone mosaic only appears on the left side of the image. Excluding those, 1437 of 11,990 cones were assigned to the smaller Cluster 1. On average (mean ± standard deviation (SD)), 12.2 ± 1.93% of cones were classified into Cluster 1. When comparing the two-component mean lifetime ${\tau }_{m}$ (mean ± SD) of the cones assigned to each cluster, Cluster 1 and 2 cones had a mean lifetime of 448.6 ± 290.3 and 437.1 ± 253.8 ps, respectively. There was no significant difference between their mean lifetimes ($p=$ 0.3673) compared using a paired two-tailed t-test. Their respective means of lifetime parameter ratios ${a}_{1}/{a}_{2}$, ${\tau }_{1}/{\tau }_{2}$, and percent ${a}_{1}$ are given in Supplementary Table S3. The difference for ${a}_{1}/{a}_{2}$ between the clusters were not statistically significant ($p=$ 0.115), while that for ${\tau }_{1}/{\tau }_{2}$ ($p<$ 0.0001) and percent ${a}_{1}$ ($p<$ 0.0001) were significant. Amongst all cones for each image, the ${\tau }_{1}/{\tau }_{2}$ ratios and percent ${a}_{1}$ did not distribute bimodally.

The percentage of cones in Cluster 1 and average phasor coordinates for each cluster across all initial exposure images of each monkey were plotted against eccentricity in Supplementary Figs. S2 and S3. A multivariate analysis of variance (MANOVA) was used to evaluate the dependence of these parameters on monkey and eccentricity. The percentage of cones in Cluster 1 had a significant interaction effect between monkey and eccentricity ($p=$ 0.006) where, for two of the monkeys, the percentage of cones in Cluster 1 increased with eccentricity. The variance of Cluster 2 $g$ coordinate and $s$ coordinate depend only on monkey. For rods, the dependence of the $g$ and $s$ coordinates on eccentricity is significant ($p=$ 0.004 and $p=$ 0.002, respectively).

### AOFLIO measurements at the same location can be acquired repeatedly

To assess the repeatability of fluorescence lifetime phasor analysis to separate cones into two clusters, 16 of the 45 initial exposure locations were randomly selected and imaged a second time with a 3 mW (~ 877 J/cm^2^) exposure (“Paradigm 1”). This included the two locations where the cones assigned to Cluster 1 appeared only in a portion of the image (locations C and D) and one location without two distinct clusters (location B), which did not differ with the second exposure. The latter image was not included in further assessing repeatability.

Supplementary Table S2 (rows labeled “Paradigm 1”) provides the number of cones marked and classified for Paradigm 1. At 15 of the 16 locations, 324 out of 3213 total cones were assigned to Cluster 1 through GMM. A further 7 cones that were visually dimmer than neighboring cones were manually assigned to Cluster 1, yielding a total of 331 Cluster 1 cones; an example of a visually dimmer cone is shown in Supplementary Fig. S4. Excluding the two locations without fully distributed Cluster 1 cone mosaics (locations C and D), 11.4 ± 2.71% of cones were classified into Cluster 1.

Comparing the identification of Cluster 1 cones between the initial exposures and the second exposures using Paradigm 1, as showcased in Fig. 1, 313 cones matched the 342 cones identified in the initial exposure. Of the cones assigned to Cluster 1 in the initial exposure, 29 were classified as Cluster 2 in Paradigm 1. Conversely, 18 cones classified as Cluster 2 in the initial exposure were classified as Cluster 1 in Paradigm 1. In both exposures, 2853 cones were assigned to Cluster 2. This represents 91.5% sensitivity and 99.4% specificity.

**Figure 3:**
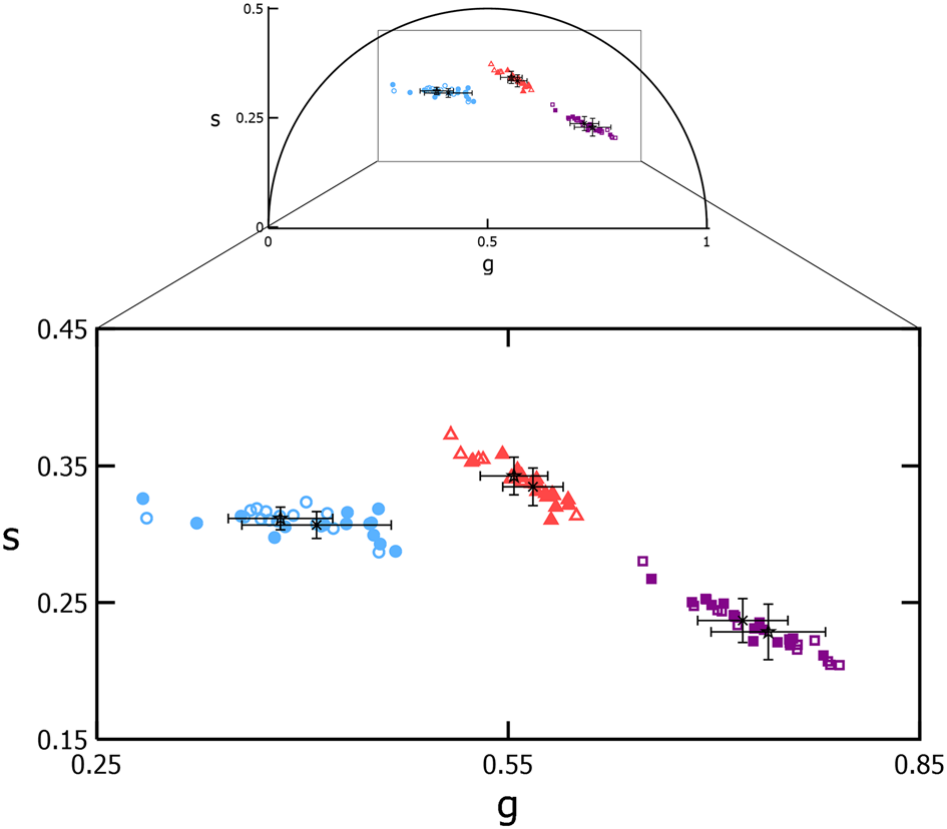
The phasor coordinates of photoreceptor classes between imaging exposures. Phasor plot with inset comparing the average phasor coordinate of the two clusters of cones (blue circles and red triangles) and one cluster of rods (purple squares) identified for 15 locations (in 3 monkeys) each imaged on two different days (filled—Day 1; open—Day 2). For each cluster, the error bars show the standard deviation of the phasor coordinates for each day (X—Day 1; star—Day 2).

A phasor plot of the average phasor coordinates of each cluster for the 15 locations is shown in Fig. 3. The initial exposure and Paradigm 1 average phasor coordinate for Clusters 1, 2, and 3 are provided in Supplementary Table S1. Unlike the initial exposures, neither the percentage of Cluster 1 cones nor phasor coordinates depended on monkey, eccentricity, or their interaction. This may be a consequence of the lower number of retinal locations tested.

In Supplementary Fig. S5, test–retest repeatability was assessed between the initial exposure and Paradigm 1 by computing the intraclass correlation coefficient (ICC) for the means of individual variables. The Cluster 1 and 2 phasors showed a poor to fair correlation for ${\tau }_{m}, g$, and $s$ ($ICC\le$ 0.53, Supplementary Fig. S5). However, Hotelling’s T^2^ test revealed that the cluster means are not significantly different (${p}_{Cluster1}=$ 0.147 and ${p}_{Cluster2}=$ 0.0863). There was a significant difference between the Cluster 3 means using Hotelling’s T^2^ test (${p}_{Cluster3}=$ 0.0421), but a better intraclass correlation ($ICC\sim$ 0.72).

### Subset of cones identified with phasor match selectively damaged S cones

Schwarz et al. determined that after exposure of the photoreceptors to 7 mW of a 730 nm femtosecond pulsed laser for 120 s (856 J/cm^2^ retinal radiant exposure), an immediate decrease in emitted florescence intensity in a subset of cones was observed^18^. These cones were later determined via histological examination to be S cones due to a substantial reduction of S cone opsin expression. Here, we implemented “Paradigm 2” which consisted of two exposures. First, we imaged the photoreceptors once at 7 mW for 120 s (2047 J/cm^2^ retinal radiant exposure) to selectively damage the S cones. After > 10 min dark adaptation, we repeated the exposure at the same location to observe the cones that became hypofluorescent.

**Figure 4:**
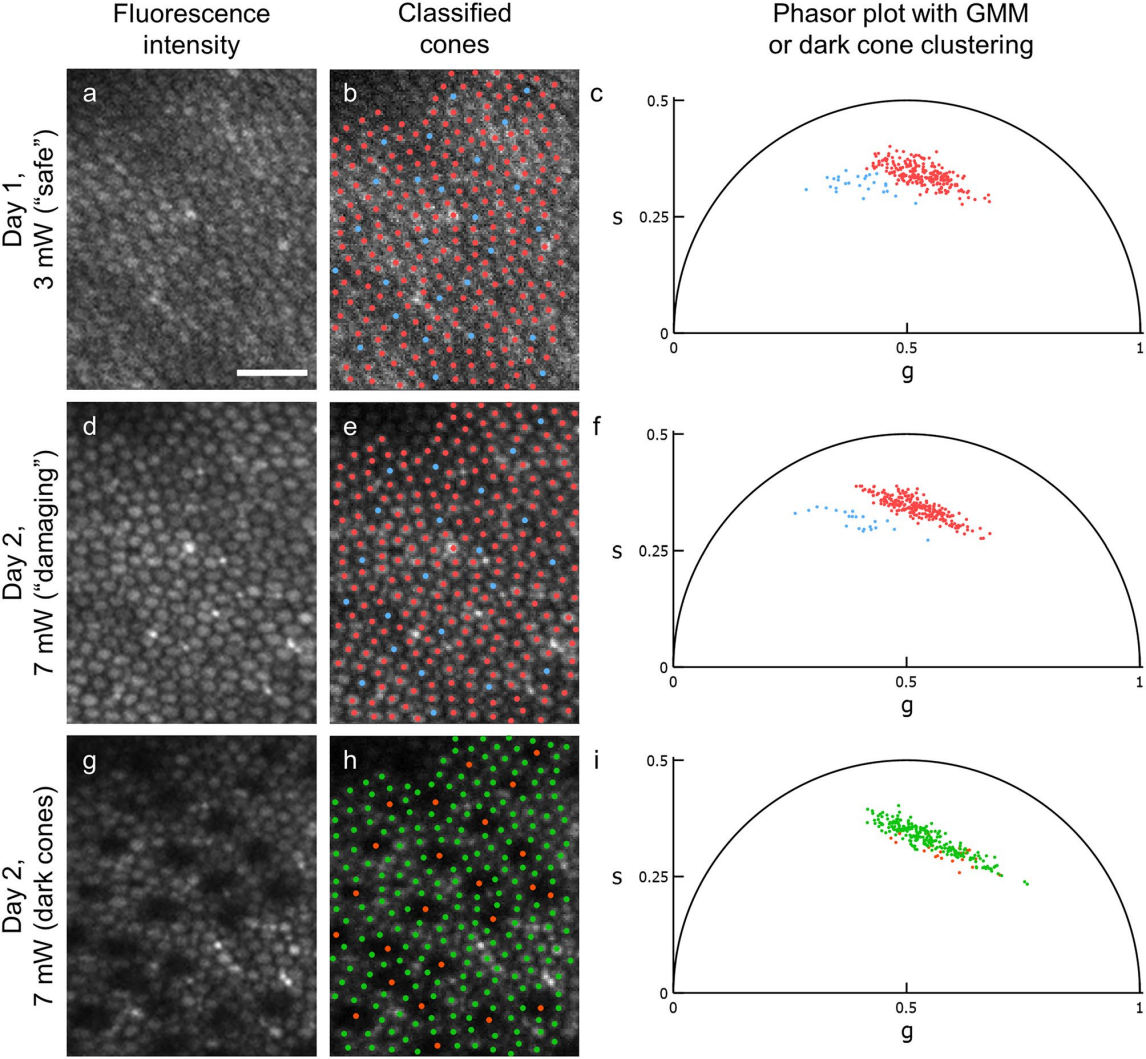
Tracking the phasor coordinates of cones before and after damage. Representative phasor data showing the correspondence between subsets of cones segregated by phasor analysis and those that eventually decrease in fluorescence intensity. An initial exposure (3 mW, 877 J/cm^2^, (**a–c**)) was performed on one day and two consecutive high-level exposures (7 mW, 2047 J/cm^2^, (**d–i**)) were performed on a subsequent day at the same location. In each fluorescence intensity image (**a,d**), the same 257 cones were marked (**b,e**) in red or blue according to their GMM cluster assignments in the corresponding phasor plots (**c,f**). 26 cones (10.1%) and 22 cones (8.7%) were assigned to Cluster 1 for (**b,e**), respectively. Each phasor coordinate represents one cone. The second Day 2 fluorescence intensity image (**g**) contains a semi-regularly spaced array of 24 cones whose intensity is noticeably lower than their neighbors. The corresponding image (**h**) and phasor plot (**i**) are marked according to whether (orange) or not (green) they decreased in fluorescence intensity. Cones obscured by the blood vessel shadow in the upper left corner of the image were not marked. Scale bar represents 20 μm.

Paradigm 2 was implemented in 11 randomly selected locations of the 45 initial exposure locations. Of those 11 locations, 7 were previously imaged under Paradigm 1. An example sequence that included an initial exposure and Paradigm 2 is shown in Fig. 4. At 10 locations, cones that were damaged by a high-level exposure (Fig. 4d–f) could be visually identified in subsequent imaging by a decrease in fluorescence intensity (Fig. 4g–i). This included one location (location C) whose initial exposure and Paradigm 1 GMM revealed Cluster 1 cones only on the top half of the image. Location B, whose initial exposure and Paradigm 1 phasors yielded only one cluster, was imaged again; the phasor plot of latter image, in agreement with previous results, yielded only one cluster and was not included in the analysis of phasor coordinates.

The first exposure of Paradigm 2 is meant to induce selective S cone damage, so there should be no evidence of visible damage in the fluorescence intensity images. Yet at 6 of the 10 locations, 60 cones (10.0 ± 3.89 cones per image) with decreased fluorescence intensity were identified. The phasor of cones that hypofluoresced shifted towards the region of remaining photoreceptors, no longer giving the appearance of two distinct clusters.

The second exposure of Paradigm 2 is meant to observe damaged cones through both fluorescence intensity and phasor analysis. At 9 of 10 locations, 257 cones were identified by eye to have decreased in fluorescence intensity relative to their neighbors as in Fig. 4g; this included the 60 cones observed in the first exposure of Paradigm 2 which remained dark. The phasor of cones that darkened here also shifted towards the region of remaining photoreceptors. At the remaining 1 of 10 locations (location E), the presence of cones with decreased intensity and two clusters in the phasor plot were observed; GMM identified for this image 24 cones assigned to Cluster 1, none of which appeared visually abnormal. Combining the cones with decreased fluorescence intensity and the 24 Cluster 1 cones in location E yielded a total of 281 cones. Between the initial exposure and second exposure of Paradigm 2, 254 cones were classified as Cluster 1 in the former and became hypofluorescent in the latter. Another 27 initial exposure cones originally classified as Cluster 2 were observed in Paradigm 2 as 12 hypofluorescent cones and 15 Cluster 1 cones. Another 31 initial exposure Cluster 1 cones were identified in the second exposure of Paradigm 2 as non-dark cones. Finally, 1957 cones were classified as Cluster 2 in the initial exposure and as non-dark in the second exposure of Paradigm 2. Thus, phasor analysis on initial exposure cones predicts the cones that decrease in fluorescence intensity as observed in Paradigm 2 s exposure with a sensitivity of 89.1% and specificity of 98.6%. Overall, 86.3% of initial exposure Cluster 1 cones turned dark compared to 0.605% of initial exposure Cluster 2 cones.

**Figure 5:**
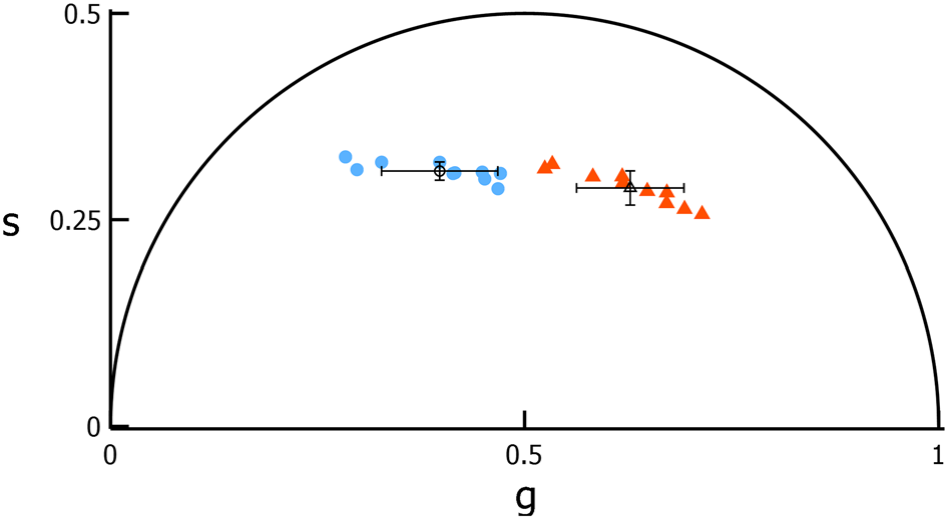
The shift in phasor identified in the smaller cone cluster after damage. Phasor plot comparing the average phasor coordinate of initial exposure Cluster 1 cones (blue circles) and Paradigm 2 s exposure cones that decrease in fluorescence intensity (orange triangles) at 10 locations in 3 monkeys.

**Figure 6:**
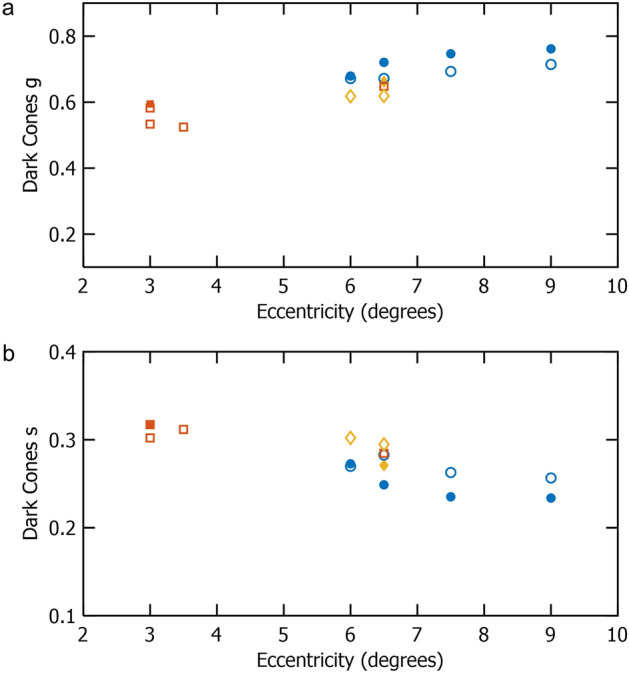
The dependence of the phasor coordinates of damaged cones on eccentricity. Plots of average phasor coordinates g (**a**) and s (**b**) versus eccentricity for images acquired with Paradigm 2 containing multiple cones that decrease in fluorescence intensity. Within each plot, a point represents one of 10 locations in 3 monkeys (blue circles—308; red squares—406; yellow triangles—605). Some cones were already damaged and were subsequently identified in Paradigm 2 first exposure (filled). Other cones were damaged as expected and identified in Paradigm 2 s exposure (open).

The average coordinates of Cluster 1 cones in the initial exposure and the second exposure of Paradigm 2 for the 10 locations are shown in Fig. 5; values are provided in Supplementary Table S1. The phasor coordinates of the second exposure of Paradigm 2 dark cones shifted rightwards relative to those initial exposure Cluster 1 cones. This indicates a shift towards shorter lifetimes. The means of the initial exposure Cluster 1 and Paradigm 2 dark cone cluster phasor coordinates are significantly different ($p<$ 0.0005). Plotting the individual $g$ and $s$ coordinates of the dark cones against retinal eccentricity (Fig. 6) reveals a variation in the shift of their phasor coordinates with eccentricity.

The mean coordinates of initial exposure Cluster 2 and the cones that remain in the second exposure of Paradigm 2, barring the Cluster 1 cones of location E, are not significantly different ($p=$ 0.0927). Coordinates are provided in Supplementary Table S1.

## Discussion

We have presented the first extensive application of the phasor method of analysis to fluorescence lifetime data of macaque photoreceptors acquired in vivo at the cellular scale. With this technique, we have demonstrated different fluorescence lifetime characteristics between photoreceptor classes, including two distinct groups of cones. This is likely associated with functional variations.

Our data are consistent with the hypothesis that S cones, M/L cones, and rods have divergent AOFLIO signatures. The cone classes correspond to Clusters 1 and 2, respectively, as classified using a Gaussian mixture model. For the initial exposures, we consistently labeled a semi-crystalline mosaic where putative S cones were rarely adjacent one another accounting for ~ 12% of all cones (Fig. 1, Supplementary Fig. S6). This percentage for data collected between 3° and 16° is similar to the percentage of S cones observed across several species of primates in the same family *Cercopithecidae* ranging from 10 to 14% beyond 1° eccentricity^20–23^. In *M. fascicularis*, the species used in this study, the S cone distribution peaks at 200 µm eccentricity (~ 1°) and decreased to 11% at 800 µm (~ 3°)^23^.

The S cone identities of these sparse cones were further corroborated by selective damage with a high-level light exposure (Paradigm 2; Fig. 4), resulting in a visually apparent decrease in their fluorescence intensity. Based on the selective S cone damage reported by Schwarz et al. from which we based Paradigm 2, we concluded that, in exposed regions, the majority of hypofluorescent cones are damaged S cones^18^. Concurrent damage to a small number of M and L cones was not ruled out. Here, the majority of hypofluorescent cones matched the Cluster 1 cones in the initial exposure with high sensitivity and specificity. This observation further supports our hypothesis that the smaller fraction of cones with a unique phasor signature are S cones. The consistency of the percentage identified, their spatial distribution, and the susceptibility to selective light damage provide evidence that S and M/L cones are separable in phasor space based on fluorescence lifetime differences.

Separation of photoreceptor types in phasor space was also repeatable. After duplicate imaging at 16 locations, S cones identified in the second exposure had high sensitivity and specificity as compared to the initial exposures (Fig. 1). For the same cone types on either day, the phasor coordinates were also similar (Fig. 3). These observations were valid when analyzing the coordinate pairs rather than the individual phasor components $g$ and $s$ individually (Fig. 3 vs. Supplementary Fig. S5). This demonstrates that the lifetime measured by AOFLIO is consistent when analyzed with phasor analysis, including for locations that are missing some (Supplementary Fig. S1) or all S cones. It is possible that undocumented exposures previously damaged the S cones at these locations. Unlike the cone phasors, the rod phasors are significantly different between the first and second exposures; this could be attributed to the methodology for marking rods that prevents matching boundaries of the same regions between different images. Nonetheless, we determined that AOFLIO measurements can be repeated on multiple occasions with consistent cell identification. It should be noted that the test–retest plots in Supplementary Fig. S5 showed the same 5 outlier images for ${\tau }_{m}$ across the three clusters. These outliers are due to poor decay curve fitting (${\chi }^{2} \sim$ 3) compared to the rest of the 27 images (${\chi }^{2} \sim$ 1). All fit parameters, such as binning kernel size, number of components, fitting algorithm, and number of iterations were kept constant between images, so we did not feel it was appropriate to change them for the outliers. The better repeatability of the phasor coordinates compared to ${\tau }_{m}$ helps demonstrate the simplicity of applying phasor analysis to AOFLIO data compared to multiexponential fitting.

There are several functional processes that can contribute to the separable and repeatable phasor clustering of photoreceptor lifetime data, especially in cones that appear visually normal. Each fluorescence lifetime decay measured in vivo is the aggregate of the decays of all contributing fluorophores at the imaging location. Though an individual fluorophore’s fluorescence decay is not dependent on intensity, the measured decay is directly affected by the relative fluorescence intensities (i.e., concentrations) of each contributor^24^. In the retina, contributing fluorophores include NADH and FAD, cofactors involved in energy transfer pathways of metabolism; and all-*trans*-retinol, believed to be the dominant time-varying fluorophore in the photoreceptor visual cycle^2,19^.

Metabolism should vary between the different photoreceptor types due to differences in mitochondrial activity^25–28^ or visual stimulation^19,29–31^. In particular, S cones have a lower concentration of mitochondria, prefer glycolysis over M/L cones, and are perhaps more resistant to metabolic shifts^11,31^. These differences in metabolism should affect the relative concentrations of NADH and FAD, altering the overall lifetime^15,32–36^.

Additionally, variable bleaching levels due to differences in spectral and photopic sensitivities between the photoreceptors^9,10,37,38^ may also be driving the differences in lifetime. Though both rods and cones possess a retinal pigment epithelium (RPE)-mediated visual cycle, regeneration of photopigment occurs faster in cones than in rods. This is likely due to the greater abundance of the various forms of retinol dehydrogenase and the complementary role to the RPE visual cycle of the alternate cone visual cycle by Müller cells in isomerizing all-*trans*-retinol into 11-*cis*-retinol^27,39^. The resultant higher concentration of retinol isomers relative to rods may then separate the phasor coordinates of cones and rods. Compared to M/L cones, S cones are also slower to respond to photopic stimulation, less sensitive to sinusoidal stimuli and background light levels, and are noisier^40^. Perhaps the distinguishable longer-lifetime phasor signature of S cones arises from their light-sensitivity properties, which affect their visual cycle and basal metabolic rates. Bleedthrough fluorescence from the RPE is unlikely since it weakly fluoresces with 730 nm excitation and the two-photon focal volume (much smaller than the length of the photoreceptors) is positioned vitreally closer to the inner segments^41^. Though none of these explanations clearly elucidate the molecular underpinnings for the unique fluorescence lifetime signature of S cones, they underscore the sensitivity of AOFLIO and phasor analysis to significant functional differences between photoreceptor classes.

The phasor method can also measure possible loss of S cone function. For the S cones that decrease in fluorescence intensity after the initial 2047 J/cm^2^ exposure (first exposure of Paradigm 2), their phasor coordinates shift towards those of the remaining photoreceptors with shorter lifetimes (i.e., towards (1,0), as shown in Fig. 5). This could be related to underlying cell damage mechanisms^42^. But in contrast to the relatively flat dependence of the phasor coordinates of normally fluorescent S cones on eccentricity (Supplementary Fig. S3a,b), those of damaged cones at higher eccentricities shift closer to (1,0) (Fig. 6). A 2.6-fold decrease in fluorescence of damaged cones^18^ might increase the influence of optical crosstalk (when photons are misassigned to neighboring pixels due to off-axis scattering or residual motion even after optical tracking) from surrounding cells on the aggregate decay curve. As the presence of rods and spacing of cones increase with eccentricity^22^, the impact of crosstalk on the phasor coordinates of dark S cones might scale with rod presence.

The retinal radiant exposure used for non-damaging AOFLIO exceeded that reported to induce S cone damage by 1.0%. Nonetheless, only 7 out of 331 (2.1%) S cones hypofluoresced as a consequence of initial AOFLIO measurements; 60 out of 271 (22.1%) S cones hypofluoresced after two low-level light exposures. We deemed these exposures acceptable for this study. The rationale for using this RRE stemmed from balancing tradeoffs in laser power, imaging field-of-view, and duration to maximize image quality and size of cones in the image. These properties affect the signal-to-noise ratio and ease of cone identification. Although we exceeded the RRE for S cone damage in Schwarz et al., AOFLIO was performed with half as much light for the same time duration and in a field-of-view that was less than half the size. Our results suggest a failure of linear reciprocity for the S cone damage reported by Schwarz et al.^18^, inconsistent with a traditional photochemical damage mechanism. Thus, care should be taken when translating potentially damaging light levels that may act through non-linear absorption.

It is unclear whether repeated exposures to an ultrafast pulsed laser causes cumulative damage to S cones. Between two consecutive low-level exposures, the phasor coordinates of S and M/L cones that remained visually intact did not change appreciably (Fig. 3). This suggests that at sufficiently low RREs, there are minimal adverse effects on cones by a pulsed laser at 730 nm. Alternatively, if cumulative damage did occur, then it perhaps occurred very gradually or did not affect pathways involving autofluorescent molecules. We cannot exclude the possibility that the S cone phasor cluster is a result of a damage mechanism or precursor rather than intrinsic molecular differences with M/L cones.

Nonetheless, AOFLIO can provide additional biomarkers of disease that are potentially more robust than fluorescence intensity alone. Cells with different function, health, and biochemical composition should have unique lifetime signatures and be represented by the disparate clusters in the phasor plot. In some instances, as in Fig. 1a,d, the relative fluorescence intensity may vary widely upon duplicate imaging at a location. As with all adaptive optics ophthalmoscopy, the image quality can vary between sessions based on factors including tear film uniformity, contact lens position, and quality of the adaptive optics correction^43^. This can impact the total number of photons collected. In other instances, bright cones, rods, or other puncta may appear, the causes of which are unclear. The advantage of AOFLIO is that fluorescence lifetime signatures are independent of overall intensity provided there is sufficient SNR for analysis^24,44^. This is often ensured through thresholding of pixels with insufficient photon counts. In this study, decay curves for all pixels within a cone or rod region were summed, so SNR was not an issue. Cones that had different relative intensities repeatably had similar lifetimes (initial exposure and Paradigm 1). It did not seem that variations in intensity between imaging sessions (Fig. 1a,d) altered the ability of phasor analysis to separate S from M/L cones and rods.

Previous analysis^8^ of photoreceptor data using biexponential fitting to extract lifetime components and calculate mean lifetimes was able to distinguish between rods and cones, but not between cone classes. There are several reasons why multiexponential fitting may not adequately parse out the unique S cone signature. The first relies on assumptions made about the number of fit components that may not be biologically driven nor correctly represent all contributing fluorophores in the tissue. Another major assumption of fluorescence lifetime is that the radiative decay profile conforms to a single- or multiexponential profile. This may be true for isolated molecular species. But in complex biological systems, molecular interactions can result in nonexponential behavior^45^ or exponential decays whose decay rate varies in time^46^. Thus, additional complexities arise from having to select a model to produce accurate fits. The phasor method is a more general method for analyzing decay curves, agnostic to non-exponential decay behavior^46^ and the number of contributing fluorophores. The only parameters needed to transform the decay curves are the instrument response function (mainly a detector property) and laser repetition rate (a source property). Even if there is uncertainty about their values, neither of these parameters directly relate to the biological system.

In this study, we established the utility of phasor analysis for adaptive optics fluorescence lifetime ophthalmoscopy. Phasor analysis granted us the ability to distinguish macaque S, M/L, and rod photoreceptors based on functional differences manifested in their fluorescence lifetimes. Furthermore, we were able to observe fluorescence lifetime changes after S cone photodamage. Additional improvements to image acquisition algorithms will improve image quality and light safety, necessary before this technology can be translated into humans. Nonetheless, future studies will look at potential strategies to glean information about metabolism, the visual cycle, and other function, including comparing phasor signatures to those of known retinal fluorophores^13,16^ and measuring phasor changes in response to monochromatic stimuli. The increased ability to identify differences between cells using AOFLIO in macaque demonstrated in this work will enable longitudinal evaluations of subcellular mechanistic changes and novel therapies for diseases where cellular metabolism is thought to be compromised. This can advance our understanding of diseases including age-related macular degeneration^47–49^, retinitis pigmentosa^50–52^, glaucoma^53–56^, myopia^57^, and Leber’s hereditary optic neuropathy^58–60^, especially in a myriad of animal models.

## Materials and methods

### Imaging system

Images were collected using a previously described adaptive optics scanning light ophthalmoscope (AOSLO)^19^ with capabilities for two-photon excitation and fluorescence lifetime imaging^8^. Briefly, an 840 nm laser diode (QFLD-850-20S, Qphotonics, Ann Arbor, MI; 30 μW) for wavefront sensing, a 796 nm superluminescent diode (S790-G-I-15, Superlum, Cork, Ireland; 180 μW) for reflectance imaging, and an ultrashort pulsed Ti:Sapphire laser (MaiTai XF-1, Spectra-Physics, Milpitas, CA; ~ 55 fs pulse duration, 80 MHz repetition rate; 3 or 7 mW) tuned to 730 nm for two-photon excitation of intrinsic fluorophores were raster scanned and imaged onto the retina. Light levels represent average power measured for a 7.5 mm pupil at the system exit pupil. Reflectance videos were acquired by a photosensor module (H7422-50, Hamamatsu Photonics K.K., Hamamatsu City, Japan) with a 2.6 Airy disk diameter (ADD) confocal pinhole which collected the backscattered, descanned light from the 796 nm superluminescent diode. Excess backscattered light from the 730 nm pulsed laser was also descanned and collected by an additional photosensor module (H7422-50) with a 1.6 ADD confocal pinhole. A real-time image-based tracking algorithm^61^, operating based on the SLO reflectance images colocalized to the same location, drove a two-axis steering mirror (S-334.2SL; Physik Instrumente, Karlsruhe, Germany) to correct for image motion due to ventilation, the cardiac cycle, and drift. To position the focus of the fs-laser, defocus was applied to the deformable mirror in the AOSLO until the photoreceptors appeared in focus in the 730 nm reflectance image.

Fluorescence originating from the retina was spectrally selected for wavelengths less than 600 nm using a dichroic mirror (ET600sp, Chroma Technology Corp., Bellows Falls, VT) and collected, without being descanned, by a hybrid photomultiplier tube (HPM-100-40, Becker & Hickl GmbH, Berlin, Germany). Two 680 nm (ET680sp, Chroma) and one 600 nm (ET600sp, Chroma) shortpass filters were mounted in front of the hybrid PMT to select the emission spectra of NADH, FAD, and all-*trans*-retinol^62,63^. Two-photon excited fluorescence lifetime data were collected using a time-correlated single-photon counting scheme: a photon correlator module (SPC-160, Becker & Hickl GmbH, Berlin, Germany) compares photons detected by the hybrid PMT to a reference pulse train from the Ti:Sapphire laser detected by a photodiode (PHD-400, Becker & Hickl GmbH). Using SPCM control software (Becker & Hickl GmbH), photons are assigned to one of 256 time bins for each pixel in a 253 × 300 pixel image determined by its arrival time with respect to the scanner outputs and reference pulse.

### Experimental protocol

#### All exposures

One female and two male macaque monkeys (*Macaca fascicularis*) were prepared for imaging as described in the Supplementary Methods. All experimental protocols were approved by and in accordance with the University Committee on Animal Resources at the University of Rochester. All methods were carried out in compliance with the ARRIVE guidelines (https://arriveguidelines.org). AOFLIO was performed by exposing a rectangular 0.73° × 0.82° region of the retina to the 730 nm pulsed laser for 120 s. Locations were between 3° and 16° eccentricity. Only one eye was imaged in each primate. The primate was dark-adapted for 10 min before each acquisition. For repeated exposures, the 796 nm reflectance channel was turned on for less than 1 min after dark adaptation so that any positional drifting could be corrected. Image scale and RREs were calculated in the same way as Morgan et al., as detailed in the Supplementary Methods^64^.

#### Initial exposure

At the beginning of each set of exposures, a single 3 mW exposure (~ 877 J/cm^2^) was delivered to the retina ($n=$ 45 locations across 3 primates). The cones were not easily resolvable at lower powers or durations. We did not observe evidence of routine damage. Immediately after imaging, 796 nm reflectance images at larger field sizes were collected for reference and navigation. None of the locations had documented previous exposures to a 730 nm pulsed beam. During subsequent imaging sessions, some locations selected randomly from the original 45 were imaged under Paradigm 1 or 2 described below.

#### Paradigm 1

During a second imaging session, a single 3 mW exposure was delivered to 16 randomly selected locations of the original 45 across 3 primates.

#### Paradigm 2

To verify the identities of S compared to M/L cones, we adopted a selective damage paradigm based on Schwarz et al. where a high RRE induces damage and hypofluorescence in a semi-crystalline subset of the cone mosaic corresponding to the S cones^18^. This S cone damage paradigm is detailed in the Supplementary Methods. At 11 randomly selected locations across 3 primates (7 of which were also imaged with Paradigm 1), two 120 s images were acquired using 7 mW exposures (2047 J/cm^2^). The first exposure selectively damaged the S cones in a way that led to hypofluorescence. The second exposure was used primarily to identify the dark, damaged S cones. The acquisition of the second image was performed > 10 min after the first to ensure a visually noticeable decrease in the fluorescence intensity of the S cones.

### Processing raw data files

SPCImage lifetime analysis software (version 7.3, Becker & Hickl GmbH) provided with the TCSPC module was used to export decay traces and estimate a synthetic instrument response function (IRF) from the files generated by SPCM. For locations with multiple exposures, the images were coaligned either manually or through a cross-correlation algorithm. The images were cropped to minimize the differences between the images due to distortion caused by rotation from the monkey’s head position or sinusoiding from the raster scan^61^. The cropping was applied to the files containing the images’ decay traces. Multiple masks with manually marked individual cones or rod regions were then generated for each image as described in the Supplementary Methods. Normally, a sinusoidal artefact induced by the raster scan that makes the images appear stretched horizontally, especially at the left and right edges, is corrected by a desining function integrated into the custom image acquisition software^61^. Because commercial SPCM software was used to acquire the fluorescence lifetime images, we could not apply this correction. Thus, in some images, cones appeared elongated depending on the cropped region.

### Calculating the phasor coordinate for each region of interest

A custom MATLAB (R2017b, The MathWorks, Inc., Natick, MA) suite used the aforementioned masks to identify and sum the decay curves belonging to each cone photoreceptor or rod region (i.e., region of interest (ROI)). Each ROI can then be represented by a single decay curve. The discrete cosine and sine Fourier transforms of each aggregate decay trace were evaluated at the laser repetition rate and deconvolved with the IRF (see Supplementary Methods for full derivation and implementation of phasor method). The resulting values were treated as a Cartesian coordinate pair $(g,s)$, also known as a phasor coordinate. The phasor coordinate for each ROI was plotted on a common axis to generate a phasor plot for the fluorescence lifetime image. Phasor coordinates for clusters were reported as mean ± SD.

### Clustering the phasor coordinates with Gaussian mixture model

To cluster the two-dimensional phasor data into two distinct cone populations, *k*-means clustering with ${k}_{coneclusters}=$ 2 was used to seed the initial covariance matrices and average cluster coordinates for a Gaussian mixture model (GMM). The GMM fits two two-dimensional orthogonal Gaussian distributions to the phasor coordinates. Each coordinate is assigned to a cluster based on the likelihood of it belonging to said cluster. Using the cluster assignments, the average $g$ and $s$ were calculated for each cluster. In some instances, ${k}_{coneclusters}=$ 3 was used since the multimodal appearance of some phasor plots resulted in misclassification of cones or GMM fits that failed to converge. After initial classification, the two clusters not corresponding to putative S cones were treated as a single cluster for all subsequent analyses. An example is shown in Supplementary Fig. S6.

### Classification of cones as S or M/L cones

The extrafoveal (> ~ 3°) distribution of S cones in the primate is estimated to be approximately 8 to 12% of all cones^21,23^. Therefore, for images whose phasor plots appear to separate into two clusters, cones classified by GMM into the smaller cluster are putatively S cones. In comparing the sensitivity and specificity of S cone identification across image sequences at the same locations, the cones identified in initial exposures as S are used as a “ground truth”. For Paradigm 2, cones that were damaged and decreased in fluorescence intensity relative to their neighbors were identified by eye.

### Multiexponential fitting of cone lifetimes

To compare the mean lifetime between classified cones, a two-component exponential function

1 $$\begin{array}{c}d\left[t\right]={a}_{1}{e}^{-\frac{t}{{\tau }_{1}}}+{a}_{2}{e}^{-\frac{t}{{\tau }_{2}}},\end{array}$$

was fit to the decay curve at each pixel for all images using SPCImage. Binning, in which the decay curves within a 5 × 5 kernel centered on each pixel are summed, was performed before exponential fitting to increase the number of photons in each decay curve. The parameters ${a}_{1}$, ${\tau }_{1}$, ${a}_{2}$, and ${\tau }_{2}$ from the exponential fit were exported. A custom MATLAB script then generated the mean lifetime

2 $$\begin{array}{c}{\tau }_{m}=\frac{{a}_{1}{\tau }_{1}+{a}_{2}{\tau }_{2}}{{a}_{1}+{a}_{2}},\end{array}$$

from the exported fit parameters. The mean lifetime file was cropped as appropriate (see Processing Raw Data Files). To calculate the lifetime for each cone, the ${\tau }_{m}$ for pixels within each cone masked as above were averaged. The ${a}_{1}/{a}_{2}$ ratio, ${\tau }_{1}/{\tau }_{2}$ ratio, and percent ${a}_{1}$ were also calculated. Average lifetimes, ratios, and percent ${a}_{1}$ for clusters were reported as mean ± SD.

### Statistical analyses

To test the dependence of phasor coordinates and percent S cones on eccentricity and monkey, a multivariate analysis of variance (MANOVA) with monkey and eccentricity as independent variables was implemented using SPSS Statistics (SPSS 27, International Business Machines Corporation, Armonk, NY) ($\alpha =$ 0.05). The difference in means between GMM-assigned clusters was compared. The test–retest repeatability of individual variables ${\tau }_{m}$, $g$, and $s$ was compared between the initial exposure and Paradigm 1 by computing the intraclass correlation coefficient^65^. Groups of phasor coordinate pairs ($g$,$s$) were compared using a two-tailed two-sampled dependent Hotelling’s T2 test, implemented in MATLAB^66^ ($\alpha =$ 0.05). A paired two-tailed two-sampled t-test was used to assess the difference in means ($\alpha =$ 0.05) between ${\tau }_{m}$ of the cones assigned to each cluster.

## Supplementary Information

Supplementary Information.

## Data Availability

FLIM data are available upon request and requires commercially available analysis software from Becker & Hickl GmbH. Custom phasor software is available upon request. KTH and JJH can be contacted for data and software.
