# Supplementary material for: Separate lifetime signatures of macaque S cones, M/L cones, and rods observed with adaptive optics fluorescence lifetime ophthalmoscopy
Source: Sci Rep. 2023 Feb 11;13:2456. doi: 10.1038/s41598-023-28877-6 (PMC9922306; doi:10.1038/s41598-023-28877-6)
Supplement: Supplementary file 1 — Supplementary Information. [file 41598_2023_28877_MOESM1_ESM.docx]

**Supplementary Information for:**

Separate lifetime signatures of macaque S cones, M/L cones, and rods observed with adaptive optics fluorescence lifetime ophthalmoscopy

Khang T. Huynh,^1,2,*^ Sarah Walters,^3,4^ Emma K. Foley,^5,1^ and Jennifer J. Hunter^6,2,1,4^

^1^Department of Biomedical Engineering, University of Rochester, Rochester, NY 14627, USA

^2^Center for Visual Science, University of Rochester, Rochester, NY 14642, USA

^3^Currently with IDEX Health & Science, West Henrietta, NY 14586, USA

^4^The Institute of Optics, University of Rochester, Rochester, NY 14627, USA

^5^Currently with The Institute of Optics, University of Rochester, Rochester, NY 14627, USA

^6^Flaum Eye Institute, University of Rochester, Rochester, NY 14642, USA

* [khuynh2@ur.rochester.edu](mailto:khuynh2@ur.rochester.edu)

**Supplementary Methods**

**Animal preparation.** One female and two male macaque monkeys (*Macaca fascicularis*), aged 7-10 years, were used in this study. All experimental protocols were approved by and in accordance with the University Committee on Animal Resources at the University of Rochester. All methods were carried out in compliance with the ARRIVE guidelines (<https://arriveguidelines.org>). Imaging sessions occurred a minimum of 1 week apart. To prepare the animals for imaging, they were anesthetized ~1 hour before an imaging session with ketamine (10-20 mg/kg) and midazolam (0.25 mg/kg). Anesthesia was maintained for up to 6 hours by isoflurane (1-5%) delivered via intubation. Monkeys were placed in a prone position on a stereotaxic device and their heads secured with a horizontal gaze. To minimize eye motion, paralysis was induced using vecuronium (40-80 μg/kg/hour) or rocuronium (200-400 μg/kg/hour) up to a 6-hour period. Mydriasis and cycloplegia were induced during the imaging session with 1-2 drops each of phenylephrine hydrochloride (2.5%) and tropicamide (1%). Corneal hydration and correction of refractive error were accomplished with a custom rigid gas permeable contact lens coated with Genteal (Alcon, Fort Worth, TX). The eye was held open with a lid speculum. After the animal was prepared for imaging, the stereotactic cart was positioned to align the animal’s pupil to the exit pupil of the imaging system. Each imaging session lasted up to 6 hours.

**Image scaling and retinal radiant exposure.** Image scale and retinal radiant exposures were calculated in the same manner as Morgan et al^1^. The fluorescence intensity images were scaled by linearly scaling the LeGrand model eye: for the imaged eye in each monkey, the focal length was approximated by dividing the average of 5-6 axial length measurements (acquired for the imaged eye using an IOLMaster (Carl Zeiss Meditec AG, Jena, Germany)) by 1.4. The scaled focal length $f_{eye}^{'}$ for each eye was then used to calculate the size of a 1° cone of light on the surface of the retina in µm per degree:

$$\begin{aligned} \frac{\mu m}{degree}=2f_{eye}^{'}\tan\left( .5 deg*\frac{\pi rad}{180 deg} \right)\#\left( S1 \right) \end{aligned}$$

The retinal radiant exposures (J/cm^2^) were calculated with an approximate macaque eye focal length of 1.5 cm for all monkeys ^1^.

**Model of S cone damage.** Schwarz et al. demonstrated that a 1.1° x 1.3° exposure to a pulsed laser at 7 mW for 120 s ($RRE=$ 856 J/cm^2^) adversely affected S cones^2^. There, the damage level was determined by titrating durations and laser powers over a fixed exposure area. ~10 minutes after this exposure, a semi-crystalline subset of cone photoreceptors underwent a 2.6-fold decrease in their two-photon excited fluorescence. These cones also became hyporeflective in the corresponding reflectance images. Days to weeks later, the same cones disappeared. At low eccentricities where rod density is low and cones are densely packed, the remaining cones were slightly laterally displaced. At high eccentricities where rod density is high and occupy spaces between cones, the remaining cones were slightly laterally displaced and rods filled in the spaces once occupied by the missing cones. Staining for S opsin at these exposed locations in an excised retina revealed an absence of S opsin compared to regions not exposed to 856 J/cm^2^ retinal radiant exposure (RRE). Schwarz et al. concluded that the cones that became hypofluorescent and subsequently disappeared were S cones. In this manuscript, we adopted a similar paradigm (Paradigm 2 in the Methods) to confirm the identity of the putative S cones identified by phasor analysis on initial exposure data. After a high RRE, cones that became hypofluorescent were identified as S cones. Because of the eventual fate of cones exposed to a high RRE, these cones are almost certainly damaged. However, the underlying cell death pathways and exact time of cell death in relation to the onset of hypofluorescence are unclear. Thus, any cones that underwent an acute, substantial decrease in fluorescence intensity after a high RRE were referred to as “damaged”.

**Manually marking individual cones.** To delineate the groups of pixels corresponding to each cone, custom masking software was used by trained graders. Each cone in the fluorescence intensity image was manually encircled by an ellipse defined by its position on the image, major and minor axes, and angle. Similar to criteria used elsewhere^3–5^, only cones whose cell boundaries were distinct to the trained eye were counted. Cones whose cell boundaries were not distinct, blurry due to poor local image quality, or obscured by a blood vessel shadow were ignored. We did not expect manual marking to significantly bias results as mostly complete and contiguous mosaics of largely uniform cones were delineated. Furthermore, graders were blinded to the identity of cones as this step is a prerequisite for cone clustering described in the main Methods. After marking, the ellipse axes were reduced between 20 and 28% so that only the central cone regions are included in the regions of interest. That range was used to ensure adequate separation between cones of different sizes and spacing across retinal eccentricity. Considering only the central cone regions in their respective masks reduces the influence of optical crosstalk on the data. For locations with multiple exposures, only those cones that were easily identified across all images were marked.

**Manually marking rod regions.** Because individual rods are much smaller and more difficult to resolve than cones^6^, non-cone portions of each image were divided into regions containing multiple rods. An inverse cone mask, with the cone regions expanded to minimize optical crosstalk on rod fluorescence, was generated. *k*-means clustering was used to partition the rod mask into $k_{rodregions}$ regions of roughly equal area. $k_{rodregions}$ was calculated so that each region had approximately the same number of photons as a single cone. Once the rod mask was generated and partitioned, regions containing blood vessels, whose shadows may obscure the identification of cones, were excluded from the final mask. Some regions along the edges of the image, where alignment was poor due to image distortion and rotation were also excluded. These issues, as well as the randomness of unseeded *k*-means clustering in image partitioning, meant the locations and numbers of valid regions in the final rod masks were different across image sequences at the same location.

**Calculating and interpreting fluorescence lifetime decay curves in phasor space.** The phasor approach to lifetime analysis is a useful tool for visualizing the spatial distributions of fluorescence lifetime decay components. The discrete real and imaginary Fourier transforms of the decay curve at each pixel are calculated and evaluated at the laser repetition rate using the following equations:

$$\begin{aligned} g_{p,q}=\left| Re \right|_{p,q}=\frac{\sum_{t=0}^{N_{bins}} d_{p,q}\left[ t \right]\cos\left( 2\pi tnf_{laser} \right)}{\sum_{t=0}^{N_{bins}} d_{p,q}\left[ t \right]} \#\left( S2 \right) \end{aligned}$$

$$\begin{aligned} s_{p,q}=\left| Im \right|_{p,q}=\frac{\sum_{t=0}^{N_{bins}} d_{p,q}\left[ t \right]\sin\left( 2\pi tnf_{laser} \right)}{\sum_{t=0}^{N_{bins}} d_{p,q}\left[ t \right]}\#\left( S3 \right) \end{aligned}$$

where $d_{p,q}[t]$ is the recorded decay curve at pixel $(p,q)$, $t$ is the time bin, $N_{bins}$ is the total number of time bins in the decay curve, $n$ is the positive integer harmonic, and $f_{laser}$ is the laser repetition rate in Hz. The values $g$ and $s$ are treated as a coordinate pair and plotted on a Cartesian plot. We refer to the coordinate pair $\left( g,s \right)_{p,q}$ as the “phasor coordinate” for pixel $(p,q)$.

To assist in the visual interpretation of phasor plot, the “universal semicircle”, an axis representing all possible lifetimes of a normalized single-exponential decays is plotted. From a single-exponential

$$\begin{aligned} d\left( t \right)=\left\{ \begin{aligned} e^{-\frac{t}{\tau}}, &x\geq0 \\ 0, &x<0 \end{aligned} \right.\#\left( S4 \right) \end{aligned}$$

the normalized Fourier transform is computed as

$$\begin{aligned} \frac{\mathcal{F}\left\{ d\left( t \right) \right\}}{\int d\left( t \right)dt}=\frac{\int_{0}^{\infty} e^{-\frac{t}{\tau}}e^{-2\pi jt\xi}dt}{\int_{0}^{\infty} e^{-\frac{t}{\tau}}dt}\#(S5) \end{aligned}$$

which yields a solution

$$\begin{aligned} \frac{\mathcal{F}\left\{ d\left( t \right) \right\}}{\int d\left( t \right)dt}=\frac{1}{1+j\omega\tau}\#\left( S6 \right) \end{aligned}$$

Here, $\omega=2\pi\xi$ and $\xi={nf}_{laser}$ where $n=$ 1. From the equation, the real and imaginary components can be separated and treated as phasor coordinates

$$\begin{aligned} \left( g,s \right)_{universal}=\left( \left| Re \right|,\left| Im \right| \right)=\left( \frac{1}{1+\omega^{2}\tau^{2}}, \frac{\omega\tau}{1+\omega^{2}\tau^{2}} \right)\#\left( S7 \right) \end{aligned}$$

By evaluating $\left( g,s \right)_{universal}$ for all $\tau\geq0$, a semicircular axis with a radius of 0.5 can be plotted. $\tau=0$ corresponds to phasor coordinate $(1,0)$, while $\tau=\infty$ corresponds to phasor coordinate $(0,0)$. For $f_{laser}=80 MHz$, $(0.5,0.5)$ corresponds to $\tau\approx1.989 ns$. Thus, decays whose phasor coordinates that lie on the semicircle closer to $(1,0)$ are comprised of shorter single-exponential lifetimes, while those whose coordinates are closer to the origin are comprised of longer lifetimes. Phasor coordinates that represent pixels with multiple contributing fluorophores, and thus a sum of multiexponential decays, will lie inside the universal semicircle.

If all the single-exponential contributors are known, the overall phasor coordinate can be computed as a linear combination of these contributors

$$\begin{aligned} \left( g,s \right)=\left( \left| Re \right|,\left| Im \right| \right)=\left( \sum_{k}^{N_{k}} \frac{a_{k}}{1+\omega^{2}\tau_{k}^{2}}, \sum_{k}^{N_{k}} \frac{a_{k}\omega\tau_{k}}{1+\omega^{2}\tau_{k}^{2}} \right)\#\left( S8 \right) \end{aligned}$$

where $k$ is the $k$th fluorophore, $N_{k}$ is the total number of contributing fluorophores, and $a_{k}$ is the fractional contribution of the $k$th fluorophore with lifetime $\tau_{k}$. These fractions can be determined by the coordinate’s distance from its single-exponential contributors on the semicircle^7–10^. It should be noted that decays whose behavior is nonexponential will have corresponding phasor coordinates outside the semicircle. In practice, interpreting phasor plots of *in vivo* data is difficult to accomplish at the molecular level because the identities of every possible fluorophore and how their phasor plots compare to pure samples *in vitro* are unknown.

**Removing the influence of the system’s instrument response on phasor coordinates.** The instrument response function (IRF) describes the overall timing precision of a time-correlated single-photon counting system. An ideal TCSPC system has an infinitely narrow excitation pulse and instantaneous measurements. However, factors such as chromatic dispersion and detector response delay the recording of a photon, broadening the IRF. Consequently, the recorded fluorescence lifetime image is a convolution of the true decay profile (sometimes referred to as the fluorescence impulse response function) of a fluorophore with its instrument response function^11^. If we use the raw decay curves detected by the TCSPC board, the resultant phasor plot will be shifted to the left and rotated: the pulse broadening exaggerates the decay constant^12,13^. Thus, it is necessary to deconvolve the detected signal before extracting any meaningful information from the data. The IRF can either be measured by calibrating to a known standard such as urea, fluorescein in sodium hydroxide, or para-terphenyl in ethanol^12^ or approximated by software^14^. For this study, we estimated the IRFs using SPCImage.

The deconvolution problem is solved in the frequency domain, using the phasor coordinates of the normalized recorded signal and normalized instrument response function to solve for the coordinates of the true signal. This effectively scales and rotates the coordinates of each phasor point. The derivation of this transform is shown below.

The recorded signal $r(t)$ is a convolution of the fluorophore’s characteristic decay $d(t)$ with the IRF of the system $h(t)$:

$$\begin{aligned} r\left( t \right)=\int_{-\infty}^{\infty} d\left( t \right)h\left( t-\tau\right)d\tau=d\left( t \right)\otimes h\left( t \right)\#\left( S9 \right) \end{aligned}$$

In Fourier space, the recorded function is represented as

$$\begin{aligned} R\left( \xi\right)=D\left( \xi\right)H\left( \xi\right)\#\left( S10 \right) \end{aligned}$$

where $R\left( \xi\right)$ is the recorded function, $D\left( \xi\right)$ is the characteristic decay, and $H\left( \xi\right)$ is the IRF.

We find the phasor coordinates by applying the cosine (real) and sine (imaginary) transforms $G[]$ and $S[]$ to the characteristic decay (which are unknown), IRF, and recorded function in Eq. S10:

$$\begin{aligned} G\left[ R \right]-jS\left[ R \right]=\left( G\left[ D \right]-j S\left[ D \right] \right)\left( G\left[ H \right]-j S\left[ H \right] \right)\#\left( S seq SupEq 11 \right) \end{aligned}$$

which expands to:

$$\begin{aligned} G\left[ R \right]-jS\left[ R \right]=G\left[ D \right]G\left[ H \right]-G\left[ D \right] j S\left[ H \right]-G\left[ H \right] j S\left[ D \right]+j S\left[ D \right] j S\left[ H \right]\#\left( S seq SupEq 12 \right) \end{aligned}$$

We can simplify the equation and then separate the real and imaginary components

$$\begin{aligned} -j S\left[ R \right]=-G\left[ D \right] j S\left[ H \right]-G\left[ H \right] j S\left[ D \right]\#\left( S seq SupEq 13 \right) \end{aligned}$$

$$\begin{aligned} S\left[ R \right]=G\left[ D \right]S\left[ H \right]+G\left[ H \right]S\left[ D \right]\#\left( S seq SupEq 14 \right) \end{aligned}$$

This enables us to establish a system of equations

$$\begin{aligned} \left[ \begin{matrix} G\left[ R \right] \\ S\left[ R \right] \end{matrix} \right]=\left[ \begin{matrix} G\left[ H \right] & -S\left[ H \right] \\ S\left[ H \right] & G\left[ H \right] \end{matrix} \right]\left[ \begin{matrix} G\left[ D \right] \\ S\left[ D \right] \end{matrix} \right]\#\left( S seq SupEq 15 \right) \end{aligned}$$

We now solve for the still unknown characteristic decay phasor coordinates

$$\begin{aligned} \left[ \begin{matrix} G\left[ D \right] \\ S\left[ D \right] \end{matrix} \right]=\left[ \begin{matrix} G\left[ H \right] & -S\left[ H \right] \\ S\left[ H \right] & G\left[ H \right] \end{matrix} \right]^{-1}\left[ \begin{matrix} G\left[ R \right] \\ S\left[ R \right] \end{matrix} \right]\#\left( S seq SupEq 16 \right) \end{aligned}$$

which simplifies to

$$\begin{aligned} \left[ \begin{matrix} G\left[ D \right] \\ S\left[ D \right] \end{matrix} \right]=\frac{1}{G\left[ H \right]^{2}+S\left[ H \right]^{2}}\left[ \begin{matrix} G\left[ H \right] & S\left[ H \right] \\ -S\left[ H \right] & G\left[ H \right] \end{matrix} \right]\left[ \begin{matrix} G\left[ R \right] \\ S\left[ R \right] \end{matrix} \right]\#\left( S seq SupEq 17 \right) \end{aligned}$$

Using Eq. S17, we can now solve for the correct phasor coordinates for the characteristic function.

In summary, the IRF applies a fixed transformation on the true phasor coordinates, which is corrected through deconvolution. The coordinates of the time-domain recorded $R$ and estimated IRF $H$ signals are calculated and then employed in a system of equations to solve for the true phasor coordinates. All phasor calculations in this study are carried out using custom MATLAB software, implementing Eqs. S2 and S3 to calculate the phasor coordinates and Eq. S17 to solve the deconvolution problem.

**Supplementary Figures**


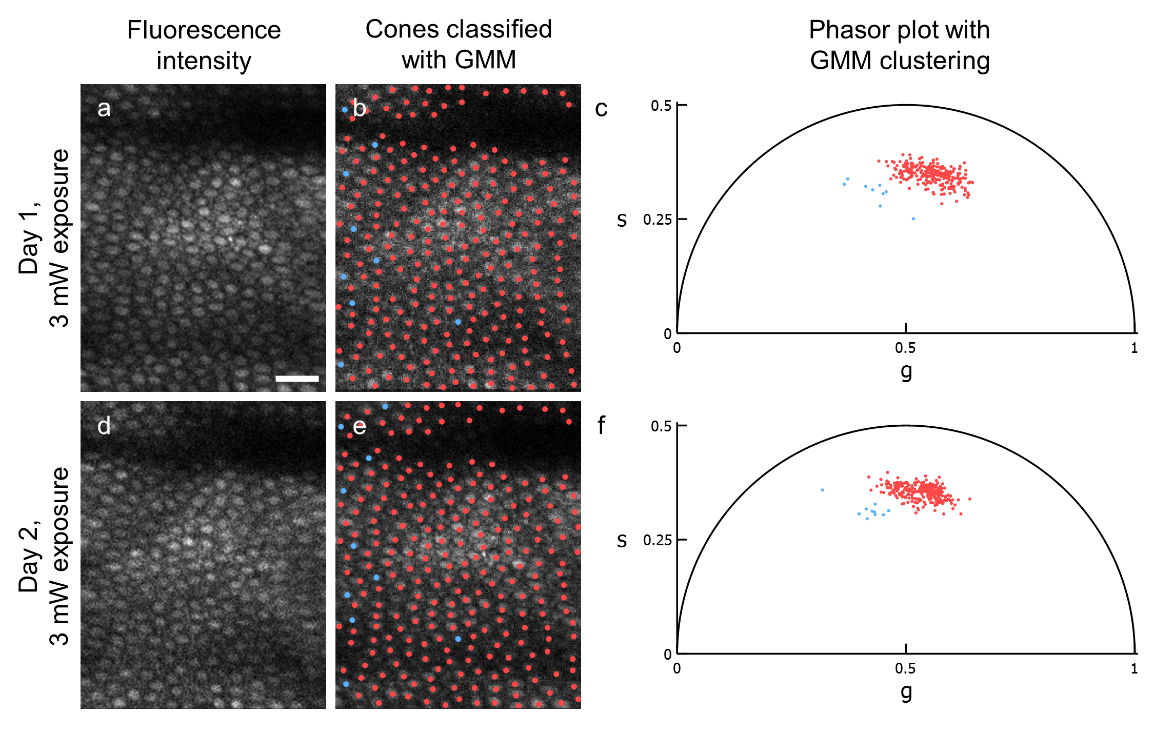


**Supplementary Figure S1.** Representative fluorescence lifetime data taken on two different days at the same location where the mosaic of cones identified as S spans only part of the image. The cones in the fluorescence intensity images (a, d) were marked (b, e) in red or blue according to their GMM cluster assignments in the phasor plots (c, f). Each phasor coordinate represents one cone. Scale bar represents 20 μm.


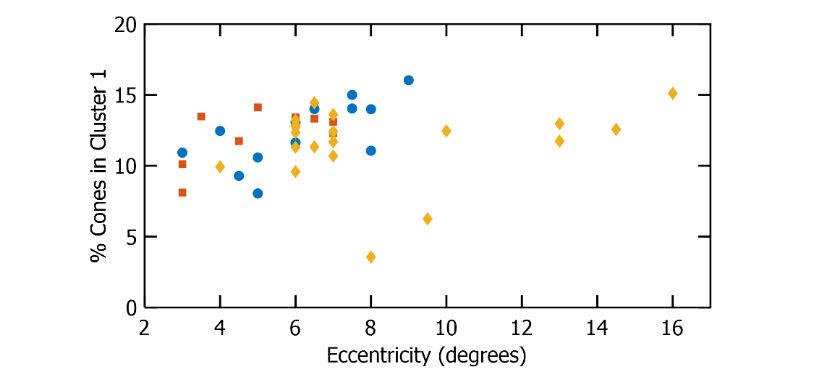


**Supplementary Figure S2.** Plot of percent cones in Cluster 1 versus eccentricity for 43 initial exposure locations in 3 monkeys (blue circles – 308; red squares – 406; yellow diamonds – 605).


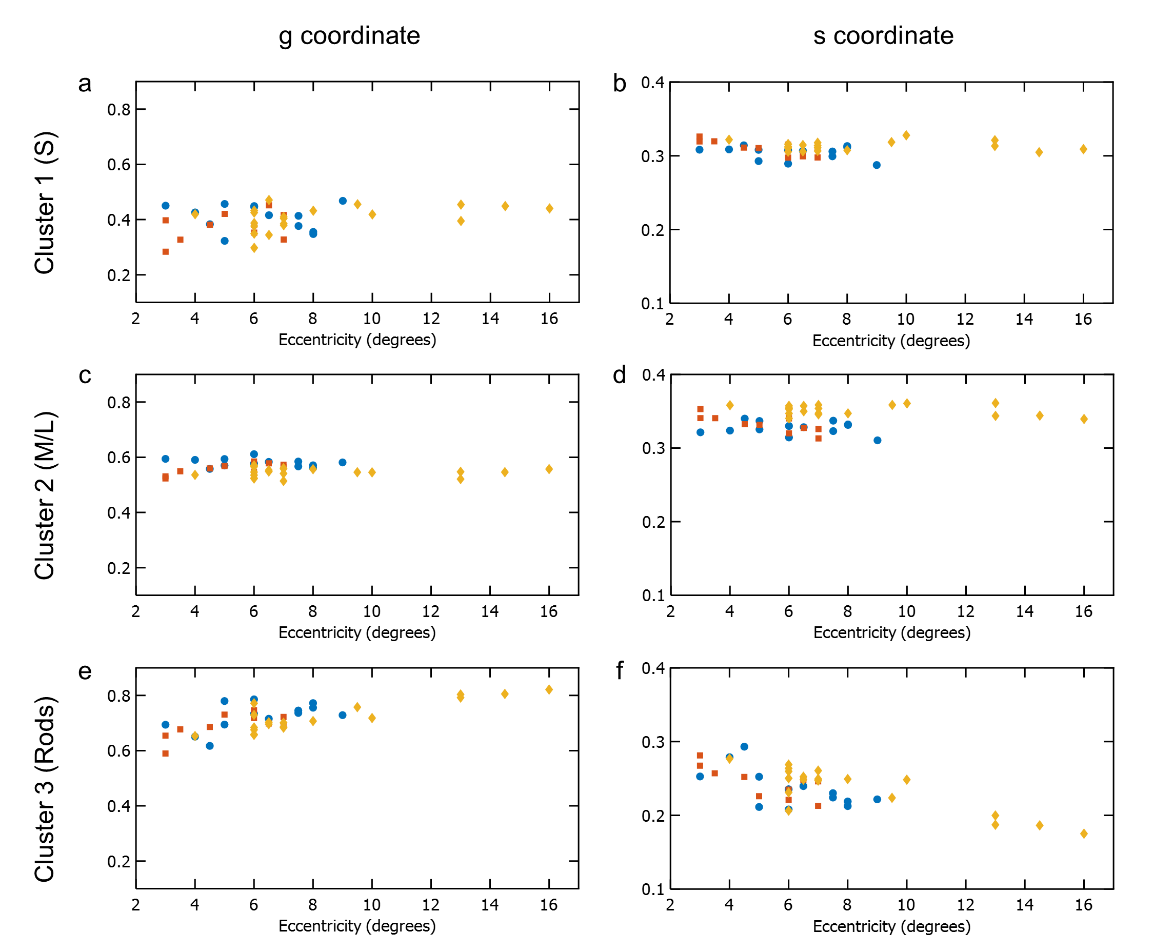


**Supplementary Figure S3.** Plots of average phasor coordinates g (a, c, e) and s (b, d, f) versus eccentricity for Clusters 1 (a, b), 2 (c, d), and 3 (e, f). Within each plot, a point represents one of 43 initial exposure locations in 3 monkeys (blue circles – 308; red squares – 406; yellow diamonds – 605).


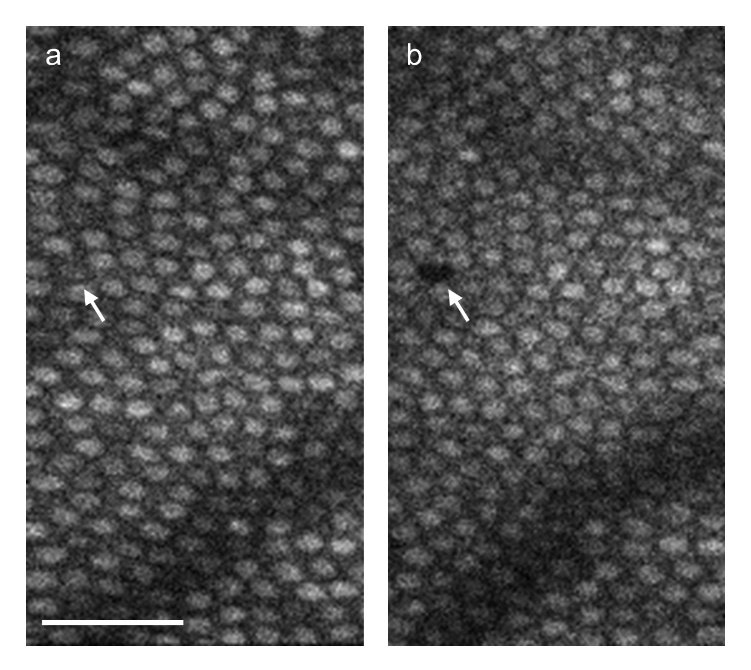


**Supplementary Figure S4.** Representative fluorescence intensity images where a cone was observed by eye to be visually dimmer than its neighbors. After an (a) initial exposure and a (b) Paradigm 1 exposure, a single cone (white arrows) decreased in fluorescence intensity. Scale bar represents 20 μm.


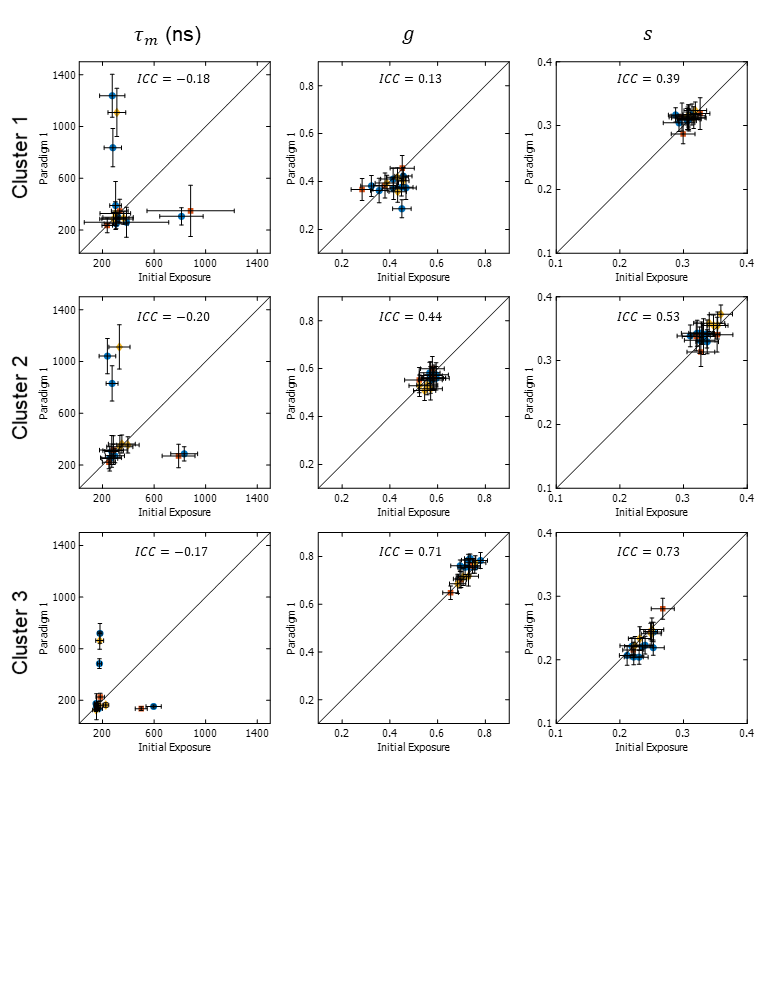


**Supplementary Figure S5.** Test-retest plots for each cluster. The variables $\tau_{m}$, $g$, and $s$ between the Initial Exposure and Paradigm 1 were compared. The intraclass correlation coefficient was calculated for each scenario for all monkeys (blue circles – 308; red squares – 406; yellow diamonds – 605).


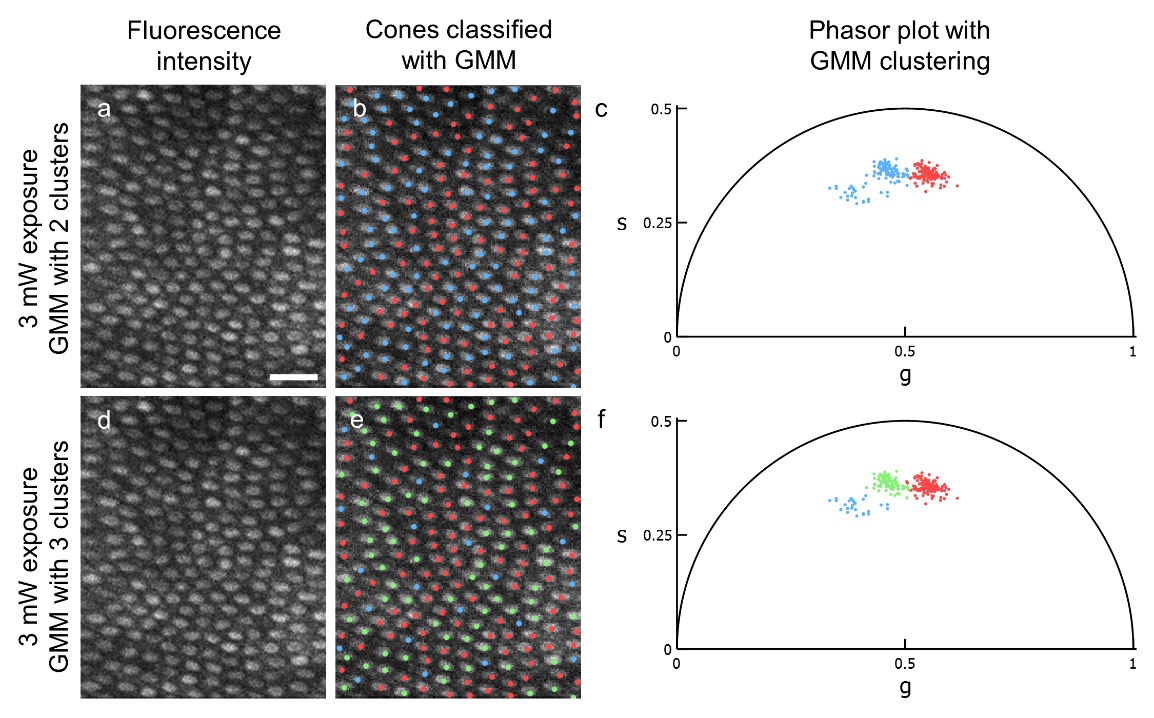


**Supplementary Figure S6.** Representative fluorescence lifetime data where the GMM classification of cones required $k=$ 3 clusters. The cones in the fluorescence intensity images (a, d) were marked (b, e) in red, green, or blue according to their GMM cluster assignments in the phasor plots (c, f). Each phasor coordinate represents one cone. The number of clusters used for GMM classification was $k=$ 2 in (a-c) and $k=$ 3 in (d-f). Out of the same 230 total cones identified in each image, 111 (48.3% of all cones) and 25 (10.9%) cones were assigned to Cluster 1, respectively. Scale bar represents 20 μm.

**Supplementary Tables**

**Supplementary Table S1.** Average phasor coordinates across all images for each imaging paradigm.

| Paradigm | Paradigm Summary | Number of locations included in average coordinate calculations | Cluster 1 Cone Phasor Coordinate (mean across n images ± standard deviation (SD)) | Cluster 2 Cone Phasor Coordinate (mean ± SD) | Cluster 3 Rod Phasor Coordinate (mean ± SD) |
| --- | --- | --- | --- | --- | --- |
| Initial exposure | Single 3 mW (~877 J/cm^2^) exposure | 43 | (0.399 ± 0.0473, 0.310 ± 0.00876) | (0.558 ± 0.0223, 0.340 ± 0.0142) | (0.715 ± 0.0509, 0.239 ± 0.0267) |
| Subset of Initial exposure locations compared to Paradigm 1 | Single 3 mW (~877 J/cm^2^) exposure | 15 | (0.410 ± 0.0545, 0.307 ± 0.00981) | (0.568 ± 0.0220, 0.335 ± 0.0138) | (0.727 ± 0.0289, 0.234 ± 0.0141) |
| Paradigm 1 | Single 3 mW (~877 J/cm^2^) exposure | 15 Initial exposure locations | (0.384 ± 0.0381, 0.312 ± 0.00829) | (0.554 ± 0.0246, 0.343 ± 0.0138) | (0.749 ± 0.0345, 0.224 ± 0.0148) |
| Subset of Initial exposure locations to compare to Paradigm 2 | Single 3 mW (~877 J/cm^2^) exposure | 10 | (0.397 ± 0.0702, 0.309 ± 0.0111) | (0.552 ± 0.0209, 0.338 ± 0.0145) | (0.691 ± 0.0448, 0.249 ± 0.0184) |
| Paradigm 2, first exposure | 7 mW (~2047 J/cm^2^) exposure | 10 Initial exposure locations | (0.414 ± 0.0324, 0.311 ± 0.00638 | (0.542 ± 0.0208, 0.338 ± 0.00739) | (0.700 ± 0.0516, 0.253 ± 0.0222) |
| Paradigm 2, second exposure | 7 mW (~2047 J/cm^2^) exposure >10 mins after (e) | 10 Initial exposure locations | (0.628 ± 0.0649, 0.288 ± 0.0207) | (0.536 ± 0.0247, 0.334 ± 0.0101) | (0.694 ± 0.0346, 0.257 ± 0.0174) |

**Supplementary Table S2.** Classification of cones for initial exposures and Paradigm 1.

| Paradigm | Number of locations | Total number of cones marked | Number of cones classified into Cluster 1 | Average number of cones classified into Cluster 1 per image (mean ± SD) | Total number of cones marked per image (mean ± SD) | Average percent of cones in Cluster 1 |
| --- | --- | --- | --- | --- | --- | --- |
| Initial exposure | 43 | 12502 | 1467 | 34.1 ± 15.0 | 290.7 ± 134.2 | 12.0 ± 2.38 % |
| Initial exposure (excluding 2 locations without fully distributed Cluster 1 cone mosaics) | 41 | 11990 | 1437 | 35.0 ± 14.7 | 292.4 ± 137.3 | 12.2 ± 1.93% |
| Paradigm 1 | 15 | 3213 | 331 | 21.6 ± 6.84 | 214.2 ± 57.7 | 10.5 ± 3.46% |
| Paradigm 1 (excluding 2 locations without fully distributed Cluster 1 cone mosaics) | 13 | 2701 | 300 | 23.1 ± 11.4 | 207.8 ± 59.5 | 11.4 ± 2.71% |

**Supplementary Table S3.** Mean lifetime parameter ratios for initial exposures.

| Paradigm | Number of locations | $\boldsymbol{a}_{\boldsymbol{1}}\boldsymbol{/}\boldsymbol{a}_{\boldsymbol{2}}$, Cluster 1 | $\boldsymbol{a}_{\boldsymbol{1}}\boldsymbol{/}\boldsymbol{a}_{\boldsymbol{2}}$, Cluster 2 | $\boldsymbol{a}_{\boldsymbol{1}}\boldsymbol{(\%)/}\boldsymbol{a}_{\boldsymbol{2}}\boldsymbol{(\%)}$, Cluster 1 | $\boldsymbol{a}_{\boldsymbol{1}}\boldsymbol{(\%)/}\boldsymbol{a}_{\boldsymbol{2}}\boldsymbol{(\%)}$, Cluster 2 | $\boldsymbol{t}_{\boldsymbol{1}}\boldsymbol{/}\boldsymbol{t}_{\boldsymbol{2}}$, Cluster 1 | $\boldsymbol{t}_{\boldsymbol{1}}\boldsymbol{/}\boldsymbol{t}_{\boldsymbol{2}}$, Cluster 2 |
| --- | --- | --- | --- | --- | --- | --- | --- |
| Initial exposure | 43 | 528.6 ± 412.4 | 440.8 ± 359.3 | 0.876 ± 0.0459 | 0.860 ± 0.0531 | 0.0934 ± 0.0646 | 0.117 ± 0.0845 |

**Supplementary References**

1. Morgan, J. I. W. *et al.* Light-Induced Retinal Changes Observed with High-Resolution Autofluorescence Imaging of the Retinal Pigment Epithelium. *Investig. Opthalmology Vis. Sci.* **49**, 3715 (2008).

2. Schwarz, C. *et al.* Selective S Cone Damage and Retinal Remodeling Following Intense Ultrashort Pulse Laser Exposures in the Near-Infrared. *Invest. Ophthalmol. Vis. Sci.* **59**, 5973–5984 (2018).

3. Morgan, J. I. W., Vergilio, G. K., Hsu, J., Dubra, A. & Cooper, R. F. The Reliability of Cone Density Measurements in the Presence of Rods. *Transl. Vis. Sci. Technol.* **7**, 21 (2018).

4. Jackson, K., Vergilio, G. K., Cooper, R. F., Ying, G.-S. & Morgan, J. I. W. A 2-Year Longitudinal Study of Normal Cone Photoreceptor Density. *Invest. Ophthalmol. Vis. Sci.* **60**, 1420–1430 (2019).

5. Walters, S., Feeks, J. A., Huynh, K. T. & Hunter, J. J. Adaptive optics two-photon excited fluorescence lifetime imaging ophthalmoscopy of photoreceptors and retinal pigment epithelium in the living non-human primate eye. *Biomed. Opt. Express* **13**, 389–407 (2022).

6. Sharma, R. *et al.* In Vivo Two-Photon Fluorescence Kinetics of Primate Rods and Cones. *Invest. Ophthalmol. Vis. Sci.* **57**, 647–657 (2016).

7. Stringari, C. *et al.* Phasor approach to fluorescence lifetime microscopy distinguishes different metabolic states of germ cells in a live tissue. *Proc. Natl. Acad. Sci.* **108**, 13582–13587 (2011).

8. Verveer, P. J. & Bastiaens, P. I. H. Evaluation of global analysis algorithms for single frequency fluorescence lifetime imaging microscopy data. *J. Microsc.* **209**, 1–7 (2003).

9. Clayton, A. H. A., Hanley, Q. S. & Verveer, P. J. Graphical representation and multicomponent analysis of single-frequency fluorescence lifetime imaging microscopy data. *J. Microsc.* **213**, 1–5 (2004).

10. Digman, M. A., Caiolfa, V. R., Zamai, M. & Gratton, E. The Phasor Approach to Fluorescence Lifetime Imaging Analysis. *Biophys. J.* **94**, L14–L16 (2008).

11. Yankelevich, D. R., Elson, D. S. & Marcu, L. Pulse sampling technique. in *Fluorescence Lifetime Spectroscopy and Imaging: Principles and Applications in Biomedical Diagnostics* (CRC Press, 2014).

12. Štefl, M., James, N. G., Ross, J. A. & Jameson, D. M. Applications of Phasors to In Vitro Time-Resolved Fluorescence Measurements. *Anal. Biochem.* **410**, 62–69 (2011).

13. Martelo, L., Fedorov, A. & Berberan-Santos, M. N. Fluorescence Phasor Plots Using Time Domain Data: Effect of the Instrument Response Function. *J. Phys. Chem. B* **119**, 10267–10274 (2015).

14. Becker, W. *The bh TCSPC Handbook*. (2021).
